# Better define beta–optimizing MDD (minimum detectable difference) when interpreting treatment-related effects of pesticides in semi-field and field studies

**DOI:** 10.1007/s11356-020-07761-0

**Published:** 2020-01-23

**Authors:** Sabine Duquesne, Urwa Alalouni, Thomas Gräff, Tobias Frische, Silvia Pieper, Sina Egerer, René Gergs, Jörn Wogram

**Affiliations:** German Environment Agency (Umweltbundesamt, UBA), Wörlitzer Platz 1, 06844 Dessau-Roßlau, Germany

**Keywords:** Ecotoxicological effects, Power analysis, Type I and II errors, Micro-/mesocosm, Lowest observed effect concentration (LOEC), No observed effect concentration (NOEC), Level of probability, Alpha and beta-values, Plant protection products, Environmental risk assessment

## Abstract

The minimum detectable difference (MDD) is a measure of the difference between the means of a treatment and the control that must exist to detect a statistically significant effect. It is a measure at a defined level of probability and a given variability of the data. It provides an indication for the robustness of statistically derived effect thresholds such as the lowest observed effect concentration (LOEC) and the no observed effect concentration (NOEC) when interpreting treatment-related effects on a population exposed to chemicals in semi-field studies (e.g., micro-/mesocosm studies) or field studies. MDD has been proposed in the guidance on tiered risk assessment for plant protection products in edge of field surface waters (EFSA Journal 11(7):3290, 2013), in order to better estimate the robustness of endpoints from such studies for taking regulatory decisions. However, the MDD calculation method as suggested in this framework does not clearly specify the power which is represented by the beta-value (i.e., the level of probability of type II error). This has implications for the interpretation of experimental results, i.e., the derivation of robust effect values and their use in risk assessment of PPPs. In this paper, different methods of MDD calculations are investigated, with an emphasis on their pre-defined levels of type II error-probability. Furthermore, a modification is suggested for an optimal use of the MDD, which ensures a high degree of certainty for decision-makers.

## Introduction

In (eco)toxicological testing, it is decisive to determine whether a specific effect has a high probability to be detected, i.e., if a certain endpoint deviation from controls at a certain concentration of a toxic substance can be identified as statistically significant effect or not. More specifically, the fact of falsely accepting or rejecting the null hypothesis (i.e., no difference between the mean of the control and the mean of a treatment: H_0_: μ_1_ = μ_2_) for a given test depends on two types of errors (the so-called type I or/and type II errors). Type I error—“false positive”—is characterized by the parameter α, which is the probability to falsely reject the null hypothesis (i.e., conclude on an effect, whereas the difference observed between the mean responses of control and treatment are due to random variability). The alternative hypothesis—the type II error, i.e., “false negative”—is characterized by the parameter β, with 1-β being the statistical power of a test (i.e., the probability of not falsely retaining the null hypothesis concludes that differences observed are due to random variability, while in reality, these are due to the treatment, whose effect remains thus undetected). Each type of errors has a different implication for the interpretation of experimental results. Type II errors are of a high relevance in ecotoxicology and particularly in the context of environmental risk assessment of toxic substances such as plant protection products (PPP) since they may lead to undetected unacceptable impacts on the ecosystems.

Several types of analyses can be performed to determine the statistical power of a test. A power analysis performed “a priori” is especially valuable for predicting the sample size needed in order to detect an effect of a given size when designing a study (ISO 2004; Ryan 2013). For standardized ecotoxicological tests, the power is defined “by design”, i.e., sample size/number of replicates (*n*) and alpha value (*α*). Standard OECD test guidelines provide usually recommendations for a priori analyses in order to determine the probability (i.e., degree of certainty) to detect a certain effect level in ecotoxicological tests. These recommendations usually set the statistical power of a test, defined as 1-β to a level of 80% (β-value of 0.2). For example, according to the OECD GD 210, fish, early-life stage toxicity test (OECD 2013) should be conducted with at least two replicates per concentration and NOEC and LOEC levels should be reported. The guideline states in section 32 that it “*is recommended that the design of the experiment and selection of statistical test permit adequate power (80% or higher) to detect changes of biological importance in endpoints where a NOEC is to be reported*.”

Power analysis can also be performed “a posteriori” (post hoc power analysis, Thomas 1997). In this case, the sample size is predetermined, and the power analysis informs us if the given test design enables to detect significant effects and with which magnitude for a specific level of certainty. In case of ecotoxicological studies, this analysis is especially useful in the case of higher tier testing methods, such as experimental ecosystems with diverse communities like micro-/mesocosm studies. In such studies, thresholds of effects such as no observed effect concentrations (NOEC) and lowest observed effect concentrations (LOEC) rather than point effect estimates such as effect concentrations (ECx) are usually derived. It is difficult to ensure a high probability of detecting small effects for such higher tier studies, i.e., to ensure a high statistical power “by design.” Indeed, field and semi-field studies are usually characterized by diverse species composition, high dynamic in population abundances over time of the represented communities, and slightly different physical and chemical boundary conditions in the replicates, typically leading to a high degree of variability within treatments in the observed data. This is also the case for long-term monitoring studies (Sims et al. 2007). In addition, micro- and mesocosm studies are usually designed with a low number of replicates due to practical constraints (e.g., increasing the number of replicates would translate in even higher cost for setup/maintenance and sample collection/processing). All these characteristics consequently have an impact on the power of the experimental study. The magnitude of detectable effects may vary—also between time-points—and can only be determined “a posteriori” and with a certain level of probability. These implications are of high importance for risk assessors who determine the suitability of the outcome of ecotoxicological studies for risk assessment decisions.

In the authorization procedures of PPP in the European Union (EU), the environmental risk assessment is performed following a tiered approach. The rather simplistic lower tier tests are performed under standardized conditions and according to specific guidelines. If an unacceptable risk is indicated, it can be then refined by conducting higher tier studies (e.g., micro-mesocosm studies). Such higher tier studies are performed under more realistic conditions but often provide more variable results than lower tier tests due to the study design and practicability constraints (i.e., natural dynamics in species assemblages usually observed over few months in only few replicates). In tier 1 assessment, the limited ability to represent field conditions and thus to describe the actual risk is addressed by associating effect values to standardized uncertainty factors (e.g., typically 10 to 100 in aquatic testing). In contrast, the underlying studies in higher tier assessment are more representative of field conditions than in tier 1, but effect values are associated to lower uncertainty factors (e.g., typically 2 to 4 in aquatic testing). Hence in higher tier testing, the statistical power should guarantee at least the same level of certainty compared with tier 1 assessment. This implies that the setting of statistical parameters such as the β-value should not be less strict in higher tier testing (with a posteriori power analysis) than in tier 1 testing (with a priori power analysis).

A specific method used in a posteriori power analyses of higher tier studies is the so-called minimum detectable difference (MDD) (Williams 1971). MDD was originally introduced as an extension of the least significant difference (LSD) concept (Snedecor and Cochran 1967), and it has been further discussed and developed in several publications afterwards (e.g., Ward et al. 1990; Loftis et al. 2001; Sanderson et al. 2009; Van der Hoeven 2008; Harcum and Dressing 2015). MDD is an important indicator that defines the difference between the means of a treatment and the control necessary to detect a statistically significant effect at a defined level of probability. The MDD analysis thus provides an indication of the robustness of the derived ecotoxicological thresholds (e.g., LOEC/NOEC) when analyzing an endpoint response at given times after treatment. As an illustration, a MDD with a β-value of 0.2 indicates the effect level that will not be overlooked in 80% of cases, i.e., at a probability of 80%. At the EU level for pesticide environmental risk assessment, MDD is first proposed in the “guidance on tiered risk assessment for plant protection products in edge of field surface waters” (also called Aquatic Guidance Document, AGD) (EFSA 2013) to provide support for interpreting outcomes from complex community studies (micro-/mesocosm studies). Although MDD informs on the sensitivity of the system towards a toxic substance and thus on its suitability for studying treatment-related effects, it is not meant to define the degree of acceptability of such studies.

Conclusions on acceptability of risks are for a large part drawn from statistical analysis of complex ecotoxicological study results. As these studies often have some shortcomings (i.e., high variability and low replicate numbers), it is of high importance to analyze and communicate the degree of certainty on which regulatory decisions are based. In this context, the regulatory decision should ensure that the probability of falsely concluding that a substance causes no effects (type II error) is minimized.

In this paper, several methods for the calculation of MDD are investigated, with an emphasis on the levels of probability related to the type II error, namely the value given to the parameter β. The implications of the β-value for the use of endpoints and related MDD values for the environmental risk assessment of PPPs are discussed. Finally, a suggestion for an optimal use of MDD for the evaluation of field and semi-field studies is proposed.

## MDD calculations for experimental studies

MDD allows reporting the difference between the means of a treatment and the control that must exist to detect a statistically significant effect in an experiment for a given endpoint at a given time and at a defined degree of certainty (probability). Thus, the concentration below the lowest concentration showing statistically significant effects (i.e., NOEC) is to be reported with the type of endpoints, e.g., NOECs for populations which are present in, e.g., micro-/mesocosm systems.

MDD Eq. 1 in AGD (EFSA 2013) was adapted from Lee and Gurland (1975):

1 $$ \mathrm{MDD}=\left({\overline{x}}_1-{\overline{x}}_2\right)=t\sqrt{\frac{s_1^2}{n_1}+\frac{s_2^2}{n_2}} $$

where ${\overline{x}}_1$ is the arithmetic mean of controls, ${\overline{x}}_2$ is the arithmetic mean of the treatment, ${s}_1^2$,${s}_2^2$ are the variance of control and treatment, *n*_1,_*n*_2_ are the numbers of control and treatment samples, *t* is the tabulated *t* value for *t* test.

It is usually expressed as percentage of control means:

2 $$ \%\mathbf{MDD}=\frac{\mathbf{MDD}\times \mathbf{100}}{\overline{{\boldsymbol{x}}_{\mathbf{1}}}} $$

The general formula of the student’s *t* value, which is used in several literatures with minor changes (e.g., Conquest 1983; Zar 1984; Oris and Bailer 1993; Sanderson et al. 2009), can be expressed as follows:

3 $$ t={t}_{\alpha, {n}_0+n-2}+{t}_{\beta, {n}_0+n-2} $$

where ${t}_{\alpha, {n}_0+n-2}$ is the student’s *t* value with (*n*_0_ + n − 2) degrees of freedom corresponding to α, ${t}_{\beta, {n}_0+n-2}$ is the student’s *t* value with (*n*_0_+ *n* − 2) degrees of freedom corresponding to β, *n*_0_ is the number of replicates for control, and *n* is the number of replicates for treatment.

The *t*-formula above (Eq. 3) could also be expressed as in Eq. 4:

4 $$ t={t}_{1-\alpha, df}+{t}_{1-\beta, df} $$

where df is the degree of freedom for α and ß, respectively.

Based on Eqs. 1 and 4, the MDD can be expressed as in Eq. 5:

5 $$ \mathbf{MDD}=\left({\overline{\boldsymbol{x}}}_{\mathbf{1}}-{\overline{\boldsymbol{x}}}_{\mathbf{2}}\right)=\left({\boldsymbol{t}}_{\mathbf{1}-\boldsymbol{\alpha}, \boldsymbol{df}}+{\boldsymbol{t}}_{\mathbf{1}-\boldsymbol{\beta}, \boldsymbol{df}}\right).\sqrt{\frac{{\boldsymbol{s}}_{\mathbf{1}}^{\mathbf{2}}}{{\boldsymbol{n}}_{\mathbf{1}}}+\frac{{\boldsymbol{s}}_{\mathbf{2}}^{\mathbf{2}}}{{\boldsymbol{n}}_{\mathbf{2}}}} $$

Equation 5 allows to control the parameters α and β. The exact derivation of this equation is presented in Ryan (2013). In practice, it allows to detect minimal changes between the control and treatment within defined levels for both type I and II errors.

The MDD equation and the use of MDD in ecotoxicological studies have been discussed in several studies. For example, Wang et al. (2000) proposed a three-step process called the minimum significant difference-based criterion testing (MSDBCT). It was suggested in that paper that when applying the MSDBCT, the values of the different parameters including α and β must be chosen by the regulators. Wang et al. (2000) further recommended to apply a β-value of 0.05 which guarantees 95% statistical power for the test to detect a difference, and consequently a high degree of certainty of not overlooking significant effects.

MDD was then further discussed, and a new approach was proposed to apply MDD to the evaluation of aquatic micro-/mesocosm studies (EFSA 2013). The MDD equation was introduced with a different variant of *t*-formula. According to Brock et al. (2015), the MDD is calculated as follows:

6 $$ \mathrm{MDD}=\left({\overline{x}}_1-{\overline{x}}_2\right)={t}_{1-\alpha, df,k}S\sqrt{\frac{1}{n_1}+\frac{1}{n_2}} $$

where *t*_1 − *α*, *df*, *k*_ is the quantile of the *t* distribution, df is the degrees of freedom for α, *k* is the number of comparisons, *S* is the residual standard error, and *n*_1,_*n*_2_ are the numbers of control and treatment samples.

The *t*-formula used in the MDD calculation according to Eq. 6 is similar to the *t*-formula (Eq. 3) used for the calculation of MDD in Eq. 1, but notably without the β-value. The *t* value would thus depend on the selected method (i.e., taken either from the general table of *t* distribution or from the table of Williams).

In Eq. (7) used in Supplemental document of Brock et al. (2015) and cited in Green et al. (2018), *t* is defined as two sided and seems to be based on the value of α and the degree of freedom (df).

7 $$ t={t}_{1-\alpha, df,k}={t}_{1-\alpha /2, df}-{\beta}_{\mathrm{Williams}}\times \left(1-\frac{1}{W}\right)/100 $$

where *W* s the *n*_0_/*n*_Treatment_*, β*_Williams_ is the weighted parameter depending on the number of treatment levels (*k*) and *α*, and tabulated in Williams (1972) for a multiple *t* test.

Hence, it is not clear which statistical power is used for the *t-*formula in MDD equation. Equation 6 indicates that either the parameter β of the *t*-formula is not specified or a default β-value of 0.5 is applied. In both cases, the probability for which a certain effect level is reported as being “detectable” is not satisfying. Indeed the equation allows to detect significant effects with only 50% certainty (50% probability of not finding significant effects which however exist), which is in conflict with the fundamental regulatory aim of avoiding to overlook considerable effects and resulting risks.

## Illustration based on aquatic mesocosm data

In order to illustrate the significance of the parameter β-value for MDD calculation and its implication for the interpretation of test results and risk assessment, respectively, data from two distinct real aquatic micro-/mesocosm studies (A and B) are presented. Both studies describe the trends observed in species assemblages after exposure to various concentrations of a toxic substance. In these studies, two insecticides “A” and “B” were used, and the most sensitive species tested were the midges *Chironomus* sp. and *Chaoborus* sp., respectively. MDD calculations were based on (i) Eq. 6 without considering the parameter β, which equals using β of 0.5, and (ii) on Eqs. (4) and (5) including Williams correcting methodology with β of 0.2.

**Fig. 1:**
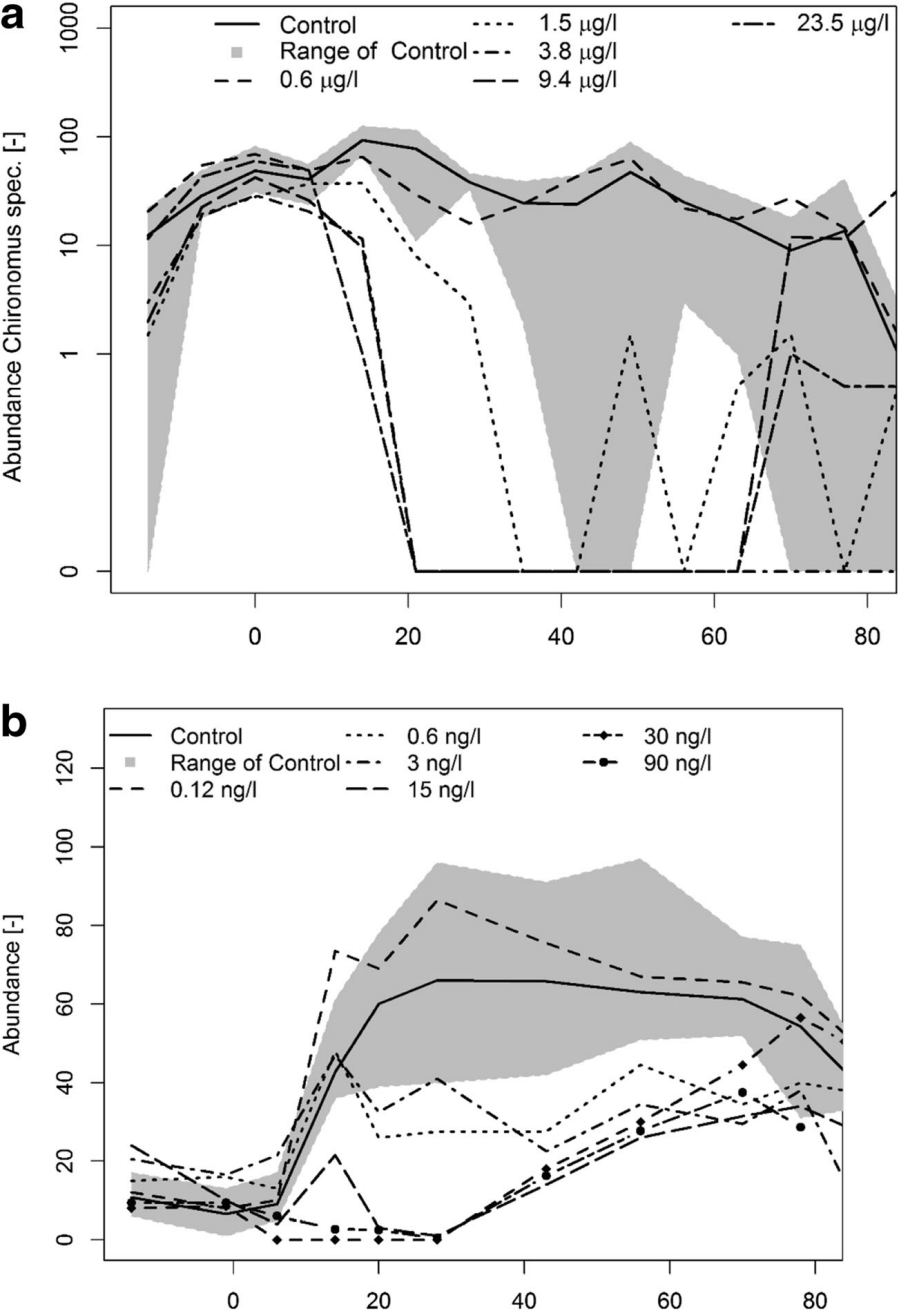
**a** Mean abundance of the adult population of *Chironomus* sp. (emergence) exposed twice (day 0 and day 21) to an insecticide “A” at five concentrations; range of the controls are represented in gray (individuals (trap*week), log-Y scale, as given in original report). **b** Mean abundance data of the sum of Chaoborus sp. (larvae and pupae) exposed twice (day 0 and day 8) to insecticide “B” at six concentrations; range of the controls are represented in gray (individuals/sample, linear Y scale, as given in original report)

In study (A), the species assemblage was treated twice with the insecticide “A” (on day 0 and day 21) at five concentrations of 0.6 to 23.5 μg/L. The study was performed with 3 replicates for the control and 2 replicates per treatment. Figure 1a represents the dynamics of the abundance of adult population of *Chironomus* sp. (emergence data). In the controls, the abundances were relatively high in the first part of the experiment, enabling a good detection of effects in treatments, but with an overall trend towards a slight decrease over time later in the post-treatment period. The treatments induced some declines in abundances of *Chironomus* sp. adults throughout the test at concentrations varying between 0.6 and 3.8 μg/L. The NOEC values varied thus from a value below 0.6 μg/L up to 1.5 μg/L, during the period of interest for deriving the effect threshold of the study (i.e., first part of the study following the 2 applications, i.e., ca day 14 to 42) and the final agreed NOEC value for risk assessment is 0.6 μg/L.

In study (B), the species assemblage was treated twice with the insecticide “B” (on day 0 and day 8) at six concentrations of 0.00012 to 0.090 μg/L. The study was performed with 4 replicates for the control and 2 replicates per treatment. Figure 1b represents the abundance dynamics of the population of *Chaoborus* sp. (sum of larvae and pupae). In the controls, the abundances were relatively low at the start of the experiment, and the overall trend was towards a clear increase over time, enabling then a good detection of effects in treatments. The treatments induced some decline in abundances of *Chaoborus* sp. at concentrations of 0.003 μg/L and 0.015 μg/L in the most relevant period after treatment, i.e., from day 14 to day 43. The NOEC values varied thus from 0.0006 to 0.003 μg/L, during the period of interest for deriving the effect threshold of the study (first part of the study following the 2 applications, i.e., ca day 14 to 43) and the final agreed NOEC value for risk assessment is 0.003 μg/L.

For statistical analysis of micro-/mesocosm results, abundance data as presented on Fig. 1a and b are usually log-transformed to approximate normal distribution and homogeneity of variance. As mentioned in Brock et al. (2015), if requirements of parametric tests are not met and rank-based tests are appropriate (e.g., the Mann–Whitney U test), other approaches such as non-parametric tests should be used. Approach such as CP-CAT which is especially suitable for Poisson distributed data such as abundances could also be considered (Lehmann et al. 2016, 2018).

For this dataset, Table 1 illustrates how the MDD values vary with the change of the parameter β-value used in *t*-formula. As MDD based on log-transformed data of abundance and represented as percentage, i.e., %MDD_log_ are difficult to interpret, it is recommended to calculate the MDD based on back-transformed abundance data, e.g., %MDD_abu_ (Brock et al. 2015).

**Table 1:** Summary of information for *Chironomus* sp. treated in study A as represented in Fig. 1a. NOEC values expressed as nominal treatment rates (in μg/L) and calculations for MDD_ln_% and MDD_abu_%, each considering 2 different beta-values (i.e., 0.5 and 0.2, respectively) and based on *n* = 3 and *n* = 2 replicates for control and treatment, respectively*

Day	0	7	14	21	28	35	42	49	56	63	70	77
Control												
Mean value of abundance	49.0	40.6	93.3	78.0	38.3	24.7	24.0	47.7	25.0	16.0	9.0	13.7
%CV	58.4	39.4	31.5	74.6	17.7	80.5	92.8	94.1	82.7	88.2	100	173
NOEC value	–	–	1.5	0.6	< 0.6	0.6	0.6	1.5	0.6	1.5	–	–
Mean value of abundance			37.5	29.5	NA	24.0	43.5	1.5	22.0	0.5		
%CV			50.9	31.2	NA	106.7	37.4	141.4	0.0	141.4		
LOEC value	–	–	3.8	1.5	0.6	1.5	1.5	3.8	1.5	3.8	–	–
Mean value of abundance			11.5	8.0	16.0	0.0	0.0	0.0	0.0	0.0		
%CV			55.3	88.4	61.9	0.0	0.0	0.0	0.0	0.0		
MDDlog% Beta 0.5	–	–	20.1	31.3	17.2	51.9	85.2	90.8	37.9	91.9	–	–
MDDlog% Beta 0.2	–	–	29.0	45.3	25.4	75.2	123	131	54.8	132	–	–
MDDabu% Beta 0.5	–	–	65.3	77.3	53.4	85.7	96.7	98.6	75.6	98.5	–	–
MDDabu% Beta 0.2	–	–	78.4	88.7	67.7	95.4	103	102	87.9	103	–	–

*These data were analyzed assuming that normal distribution and homogeneity of variance were approximated as claimed in the original dataset

**Fig. 2:**
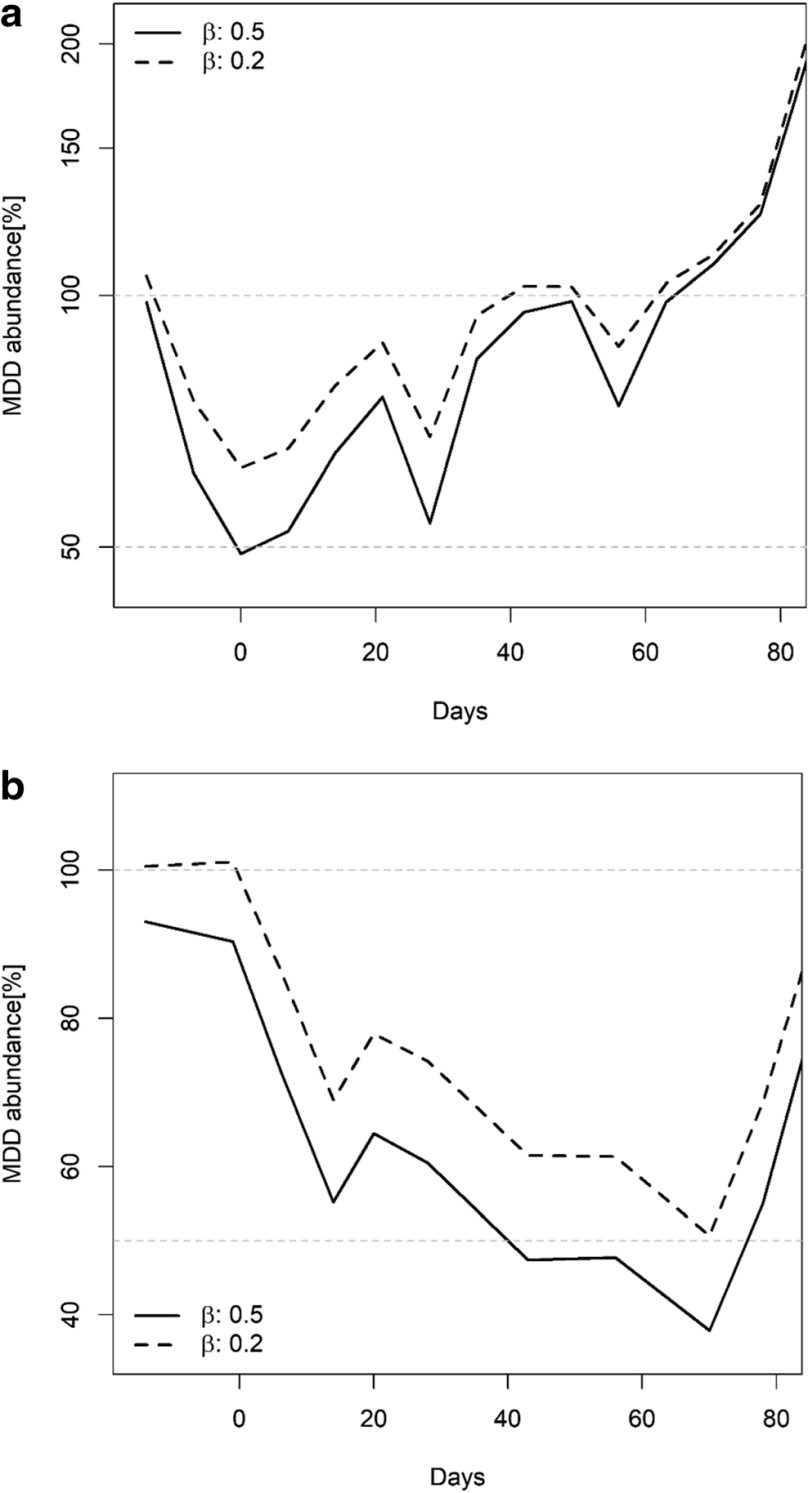
Study A. Percentage MDD values of abundance data (back transformed) of the adult population of *Chironomus* sp. for all treatments based on the mean abundance values of the control data by taking a type II error β of 0.2 and 0.5 into account. Study B. Percentage MDD values of abundance data (back transformed) for the sum of Chaoborus sp. for all treatments based on the mean abundance values of the control by taking a type II error β of 0.2 and 0.5 into account

**Fig. 3:**
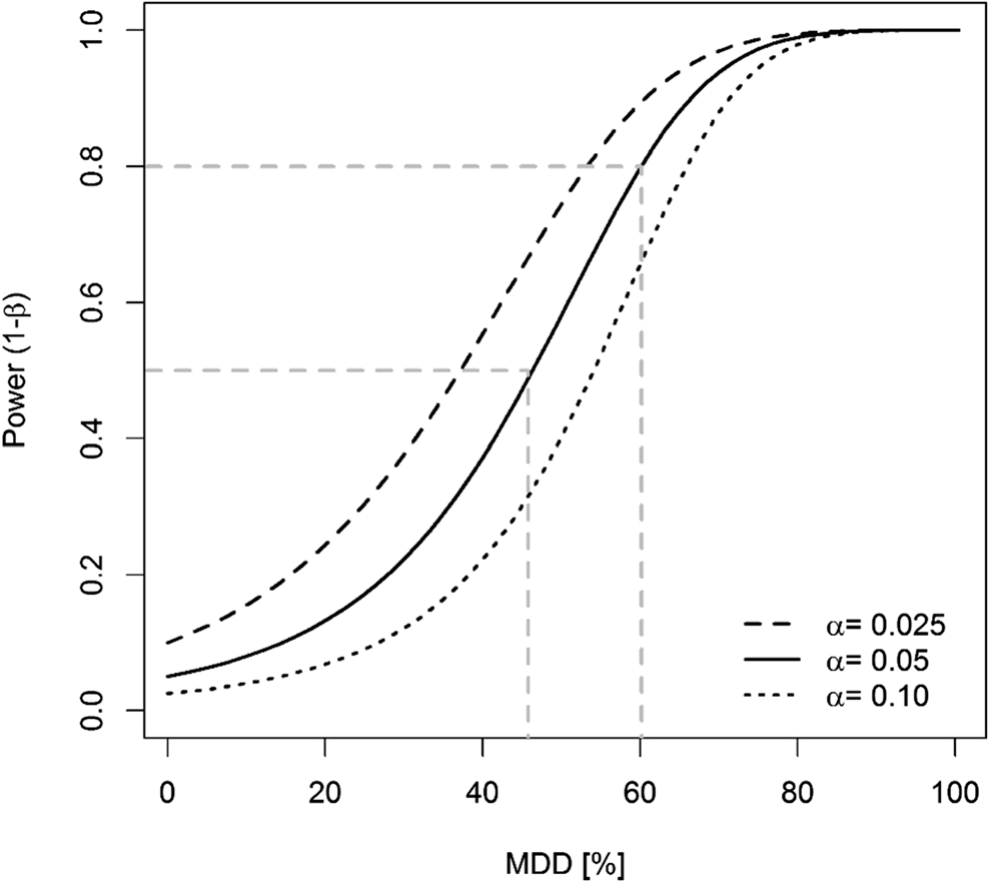
Relationship between statistical power and the minimum detectable difference (MDD in %) for three different scenarios of the type I error α (0.025, 0.05, and 0.10), calculated with the dataset of *Chironomus* sp. as given in Fig. 1a

Figure 2a and b (and Table 1) shows that during the post-treatment periods, the values of %MDD_abu_ are higher when the parameter β is set to a value of 0.2 (i.e., probability of 80%) than when set to a value of 0.5 (i.e., probability of 50% or not defined probability). In other words, when the probability of making a type II error is decreased to 20%, the value of %MDD_abu_ is increased. This is illustrated in study A, which shows that setting the parameter β to a value of 0.2 produced %MDD_abu_ values varying between 67.6 and 95.4% in the period of 14 to 35 days. These values were higher than the %MDD_abu_ values calculated when setting β to a value of 0.5 (i.e., between 53.4 and 85.7%; see Table 1). For instance, at day 21 of the study A (Fig. 2a and Table 1), the %MDD_abu_ is 77.3% when β is set to a value of 0.5 indicating that (i) only effects between control and LOEC treatment larger than 77.3% are detectable, and (ii) the probability of being correct—i.e., that these effects are not overlooked—is only of 50%. When β is set to a value of 0.2, the %MDD_abu_ increases to 88.6% (i.e., only effects larger than 88.6% are reported as being detectable), but the certainty of detecting existing effects is increased to 80%. These trends are also shown in study B (Fig. 2b). The principal relationship between power and MDD is given in Fig. 3 which indicates that in order to detect a minimum difference (MDD) of 47% for the species *Chironomus riparius*, the conclusions might be incorrect with a probability of 50% (under the conditions of study A, as shown in Table 1). In order to reach a more certain outcome (i.e., increasing the probability from 50 to 80%), the MDD value is then increased to 60%.

**Fig. 4:**
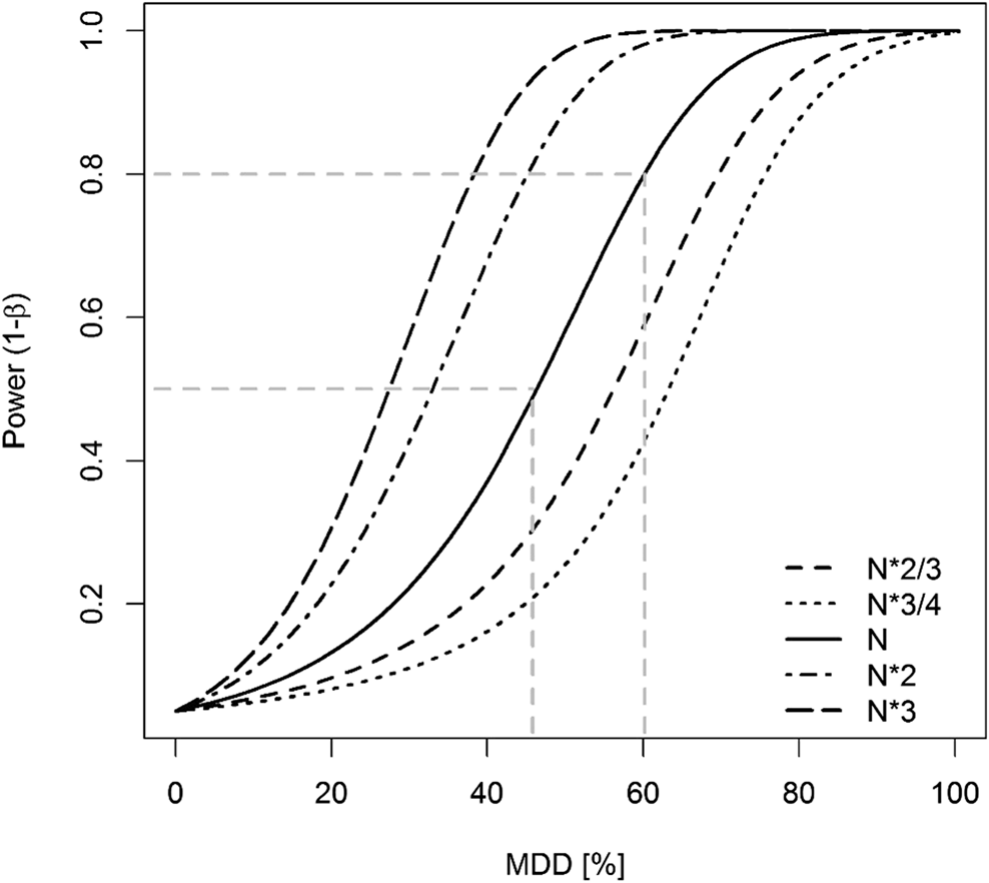
Relationship between statistical power and the minimum detectable difference (MDD in %) for five different sample size scenarios (observed N, 2/3 and 3/4 of observed N, double and triple N), calculated with the dataset of *Chironomus* sp. as given in Fig. 1a

**Fig. 5:**
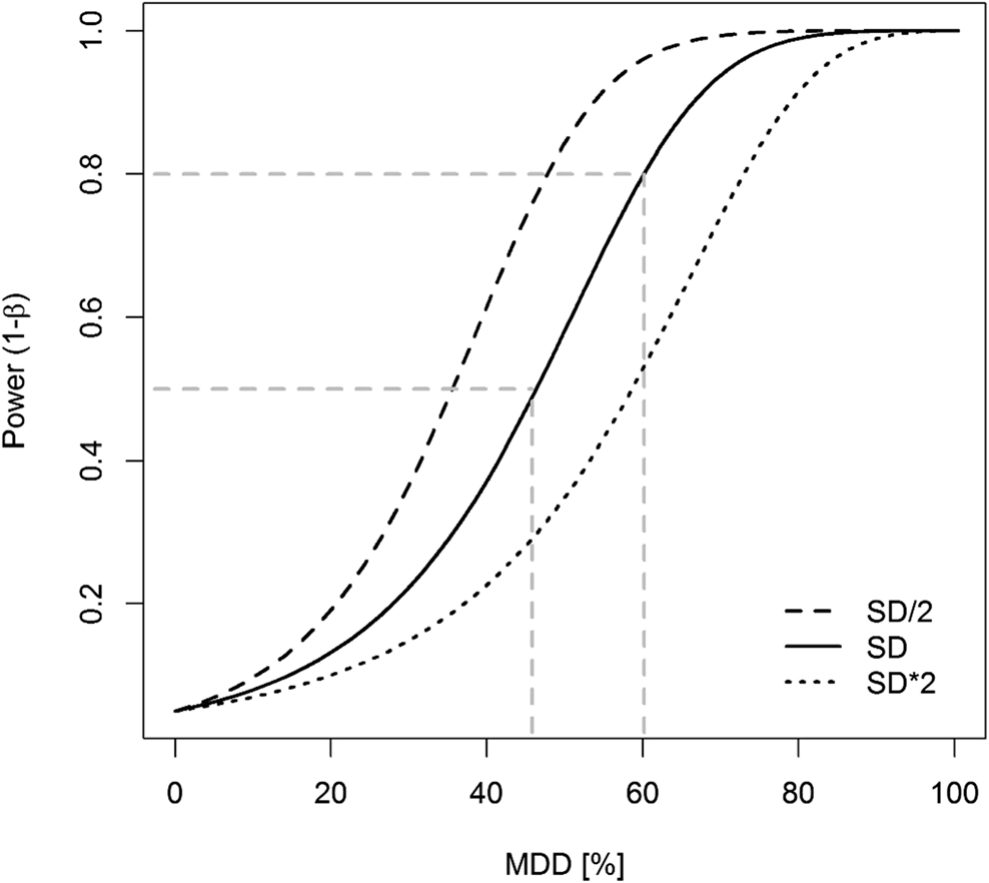
Relationship between statistical power and the minimum detectable difference (MDD in %) for three different statistical dispersion scenarios (half, estimated, and double) of the standard deviation (SD) (based on the dataset of *Chironomus* sp. as given in the Fig. 1a)

Since the statistical power (1-β) depends on the parameters alpha (α), sample size (*n*), and standard deviation (σ), changing the value of one of those parameters will result in changing the statistical power (Hanson et al. 2003; Quinn and Keough 2002). Figures 3, 4, 5 illustrate the relationships between statistical power and MDD according to those parameters; these examples are based on the dataset of *Chironomus* sp. as given in Fig. 1a. They show that (i) for fixed *n* and σ, a smaller α-value is related to a lower statistical power (at fixed MDD values) or to higher MDD values (if 1-β is fixed; Fig. 3), (ii) for fixed α and σ values, a larger sample size (i.e., *n* = number of replicates) is related to a higher statistical power (at fixed MDD values) or to smaller MDD values (if 1-β is fixed; Fig. 4), and (iii) for fixed α and n values, a larger σ-value is related to a lower statistical power (at fixed MDD values) or to higher MDD values (if 1-β is fixed; Fig. 5).

Depending on the size of effects that should be detectable and on the required degree of certainty, a high statistical power will not necessarily require an impractically large sample size (Ryan 2013), as the values of α and β could be chosen independently and regardless of *n*. Thus, the low statistical power proposed in some *t*-formulas should be avoidable, and the %MDD value could no longer imply a high degree of uncertainty (Spooner et al. 2011).

## Implications for risk assessment and recommendations

The analysis of available information and the presented case studies indicate that the method for the calculation of minimum detectable differences (MDD) could differ considerably depending on the chosen statistical parameters. The differences are linked to the formula used and the value of its parameters, especially the parameter for the type II error (i.e. β-value). Hence, the used *t*-formula should be scrutinized in order to avoid inconsistencies in the interpretation of the calculated MDD values as they eventually would have consequences in regulatory decision-making. Indeed a type I error could lead to an overly conservative regulatory decision, e.g., an unjustified refusal of authorization of the product. A type II error, however, could lead to under-protective regulatory decisions, e.g., authorizing a pesticide that has potentially severe consequences for the environment or missing to set specific conditions of use for risk mitigation. The precautionary principle in the EU chemical policy and legislation stipulates such false negative conclusions should be avoided in the environmental risk assessment (EC 2009). This implies that a suitable high level of statistical power in the a posteriori power analysis should be demanded; these analyses should be parameterized in order to report the minimum level of effect that can be actually statistically detected with a high probability.

Power analysis for lower tier standard tests and higher tier complex studies in principle address the same regulatory questions. They should thus share as much as possible similar criteria (e.g., same values of α and same values of β), in order to guarantee a similar level of certainty. We suggest therefore to align the parameter values to those used in established a priori methods (i.e., setting type II error/β to 0.2). This would ensure that the MDD analysis used as a posteriori analysis of data from higher tier studies enables regulators to avoid overlooking actual effects and taking deficient regulatory decisions.

An increase in the statistical power (1-β) can then be related to an increase of the MDD values, as illustrated in this paper. Such an increase of MDD values can have implications in the interpretation of the study outcomes. For example, higher MDD values are attributed to lower MDD classes in the classification proposed in the AGD (EFSA 2013). The MDD of most relevant endpoints should ideally exceed class II, i.e., MDD values should ideally be lower than 70%, although endpoints with lower MDD classes (I–II) (i.e., MDD values between 70 and 100%) may, however, be considered relevant. However, it should be pointed out that there is no scientifically derivable general limit for the MDD which would consider ecotoxicological tests as unsuitable (Environment Canada 2005; Brock et al. 2015; Wang et al. 2000).

It should be also noted that other methods than MDD have been proposed as statistical evaluation of higher tier studies (e.g., Lehmann et al. 2016, 2018). If appropriate statistical analysis indicates that data are not complying with established demands, the suitability of the study will be limited. But by means of an expert knowledge-based ecological evaluation of the results, the study may however contribute to a scientifically sound regulatory decision.
